# Supplementary material for: Consequences of telomere dysfunction in fibroblasts, club and basal cells for lung fibrosis development
Source: Nat Commun. 2022 Oct 6;13:5656. doi: 10.1038/s41467-022-32771-6 (PMC9537293; doi:10.1038/s41467-022-32771-6)
Supplement: Supplementary file 1 — Supplementary Information [file 41467_2022_32771_MOESM1_ESM.pdf]

Supplementary information for

Consequences of telomere dysfunction in fibroblasts, club and basal cells for lung fibrosis development

Sergio Piñeiro-Hermida[1], Paula Martínez[1,#], Giuseppe Bosso[1,#], Juana María Flores[2], Sarita Saraswati[1], Jane Connor[3], Raphael Lemaire[3], Maria A. Blasco[1,*]

[1]Telomeres and Telomerase Group, Molecular Oncology Program, Spanish National Cancer Centre (CNIO), Melchor Fernández Almagro 3, Madrid, E-28029, Spain.

[2]Animal Surgery and Medicine Department, Faculty of Veterinary Science, Complutense University of Madrid, Madrid, Spain.

[3]Research and Early Development, Early Respiratory and Immunology, BioPharmaceuticals R&D, AstraZeneca, Gaithersburg, Maryland

[#]These authors contributed equally: Paula Martínez, Giuseppe Bosso

Files included:

Supplementary Fig. 1 Female mice with deletion of *Trf1* in club cells exhibited lower lung inflammation and absence of airway remodeling.

Supplementary Fig. 2 Female mice with deletion of *Trf1* in lung basal cells exhibited lower lung inflammation and absence of airway remodeling.

Supplementary Fig. 3 Deletion of *Trf1* in embryonic basal cells decreases survival and increases airway remodeling postnatally.

Supplementary Table 1. Primer sets used for qPCR

Supplementary methods

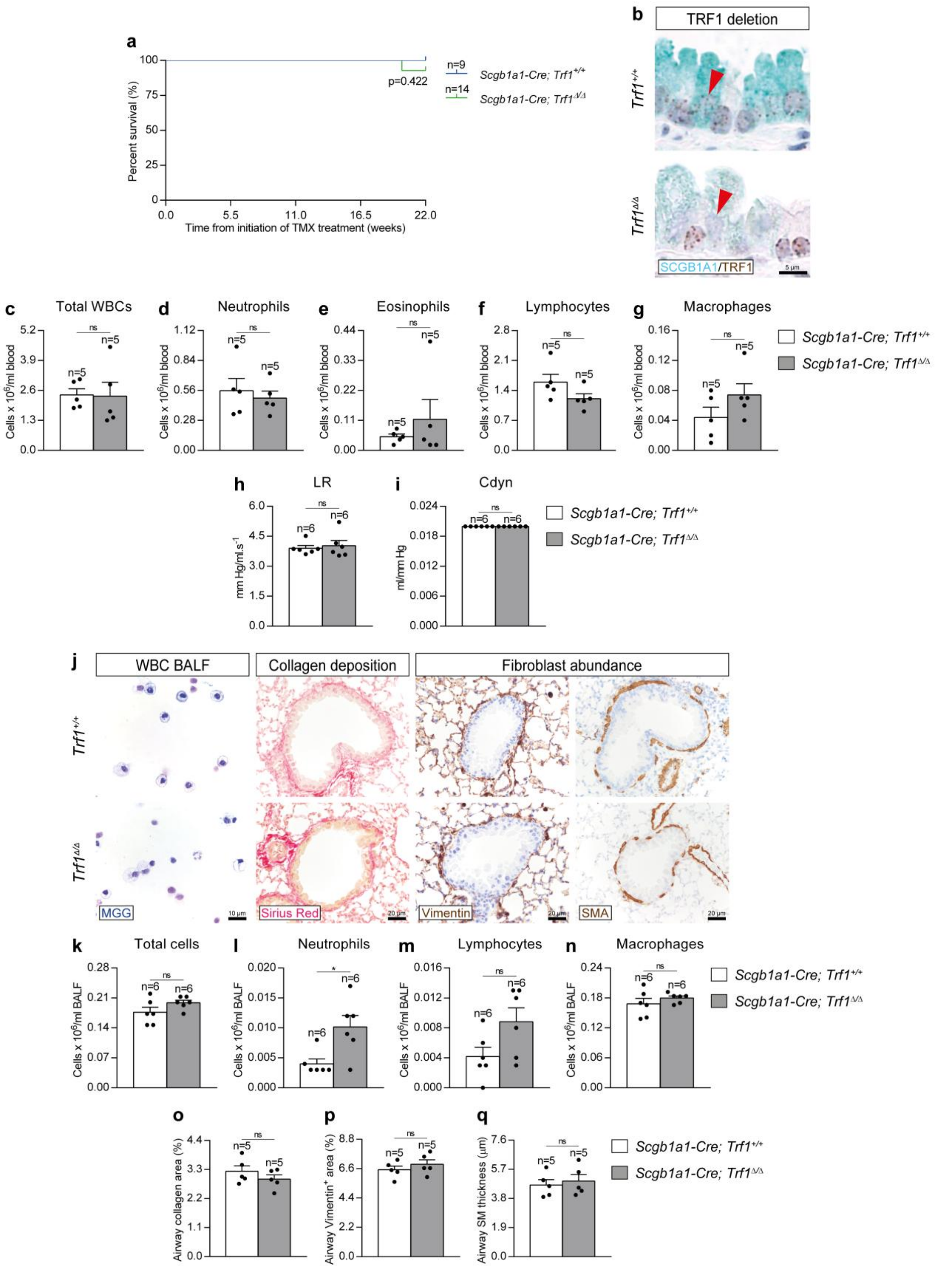

Supplementary Fig. 1 Female mice with deletion of *Trf1* in club cells exhibited lower lung inflammation and absence of airway remodeling. a Kaplan-Meier survival curves of *Scgb1a1-Cre; Trf1*$^{+/+}$ (*Trf1*$^{+/+}$) and *Scgb1a1-Cre; Trf1*$^{\Delta/\Delta}$ (*Trf1*$^{\Delta/\Delta}$) female mice. **b** Representative immunostainings for SCGB1A1 (blue) and TRF1 (brown) (red arrowheads indicate SCGB1A1^{+} club

cells with deletion of TRF1) in lung sections from *Trf1*$^{+/+}$ (*Scgb1a1-Cre; Trf1*$^{+/+}$) and *Trf1*$^{Δ/Δ}$ (*Scgb1a1-Cre; Trf1*$^{+/+}$) female mice. **c-g** Quantification of total white blood cells **(c)**, neutrophils **(d)**, eosinophils **(e)**, lymphocytes **(f)** and macrophages **(g)** in peripheral blood from *Trf1*$^{+/+}$ and *Trf1*$^{Δ/Δ}$ mice. **h-i** Lung resistance (LR) **(h)** and dynamic compliance (Cdyn) **(i)** evaluated by plethysmography in *Trf1*$^{+/+}$ and *Trf1*$^{Δ/Δ}$ mice. **j** Representative BALF cytospin preparations (May-Grünwald Giemsa (MGG)), Sirius Red staining and Vimentin and SMA immunostainings (airways) in lung sections from *Trf1*$^{+/+}$ and *Trf1*$^{Δ/Δ}$ mice. **k-q** Quantification of total **(k)** and differential BALF cell counts for neutrophils **(l)**, lymphocytes **(m)** and macrophages **(n)**, and quantification of airway collagen (Sirius Red) **(o)** and Vimentin **(p)** positive areas (%), and airway smooth muscle (SM) thickness (SMA) (µm) **(q)** in *Trf1*$^{+/+}$ and *Trf1*$^{Δ/Δ}$ mice. Data are expressed as mean ± SEM (the number of mice is indicated in each case). *$p<0.05$ (Mann-Whitney or unpaired t tests). Animal survival was assessed by the Kaplan-Meier analysis, using the log Rank (Mantel-Cox) test. Source data are provided as a Source Data file.

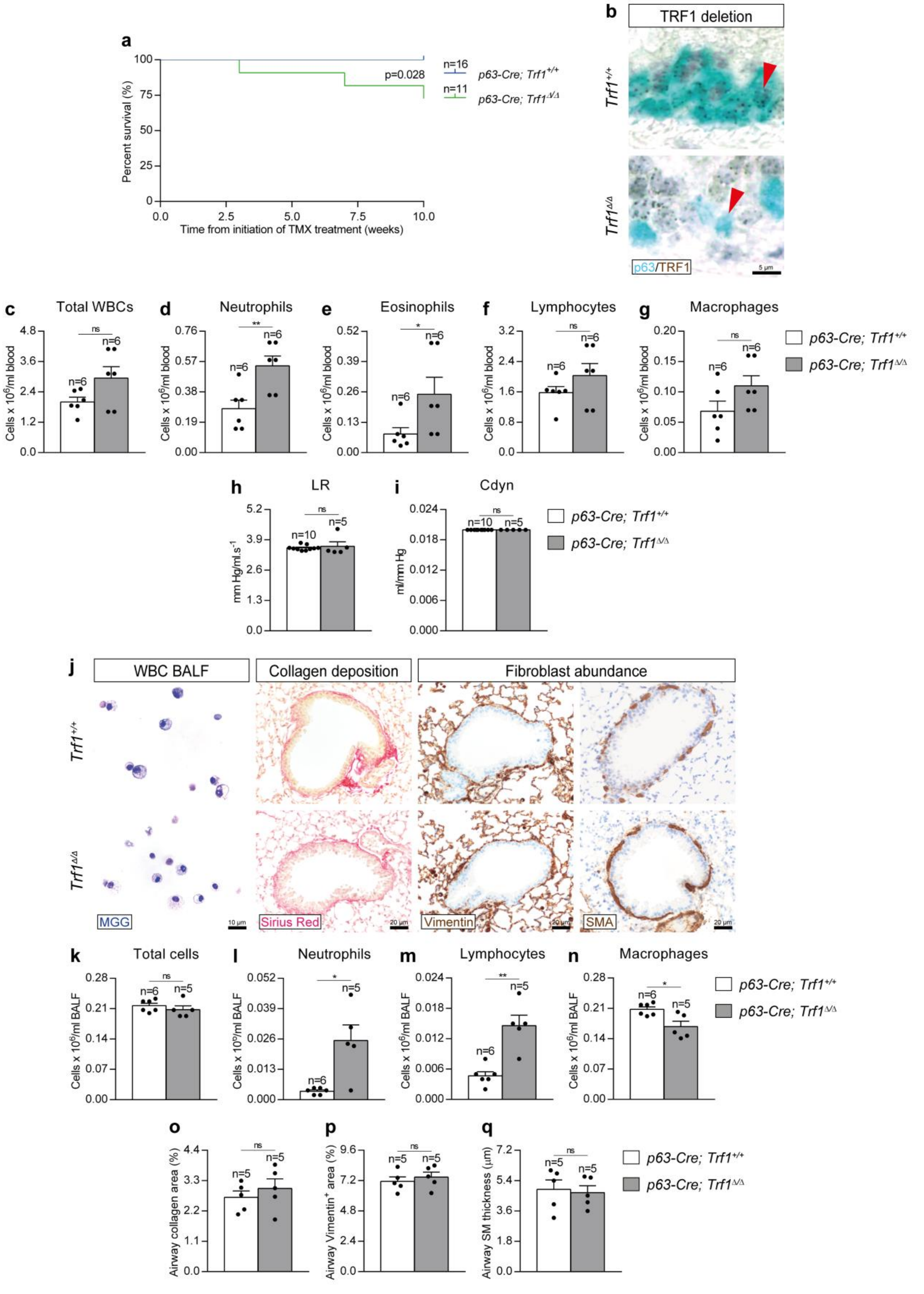

Supplementary Fig. 2 Female mice with deletion of *Trf1* in lung basal cells exhibited lower lung inflammation and absence of airway remodeling. a Kaplan-Meier survival curves of *p63-Cre; Trf1*$^{+/+}$ (*Trf1*$^{+/+}$) and *p63-Cre; Trf1*$^{\Delta/\Delta}$ (*Trf1*$^{\Delta/\Delta}$) female mice. **b** Representative immunostainings for p63 (blue) and

TRF1 (brown) (red arrowheads indicate p63$^+$ basal cells with deletion of TRF1) in lung sections from *Trf1*$^{+/+}$ (*p63-Cre; Trf1*$^{+/+}$) and *Trf1*$^{\Delta/\Delta}$ (*p63-Cre; Trf1*$^{+/+}$) female mice. **c-g** Quantification of total white blood cells **(c)**, neutrophils **(d)**, eosinophils **(e)**, lymphocytes **(f)** and macrophages **(g)** in peripheral blood from *Trf1*$^{+/+}$ and *Trf1*$^{\Delta/\Delta}$ mice. **h-i** Lung resistance (LR) **(h)** and dynamic compliance (Cdyn) **(i)** evaluated by plethysmography in *Trf1*$^{+/+}$ and *Trf1*$^{\Delta/\Delta}$ mice. **(j)** Representative BALF cytospin preparations (May-Grünwald Giemsa (MGG)), Sirius Red staining and Vimentin and SMA immunostainings (airways) in lung sections from *Trf1*$^{+/+}$ and *Trf1*$^{\Delta/\Delta}$ mice. **k-q** Quantification of total **(k)** and differential BALF cell counts for neutrophils **(l)**, lymphocytes **(m)** and macrophages **(n)**, and quantification of airway collagen (Sirius Red) **(o)** and Vimentin **(p)** positive areas (%), and airway smooth muscle (SM) thickness (SMA) (µm) **(q)** in *Trf1*$^{+/+}$ and *Trf1*$^{\Delta/\Delta}$ mice. Data are expressed as mean ± SEM (the number of mice is indicated in each case). *p<0.05*, p<0.05 (Mann-Whitney or unpaired t tests). Animal survival was assessed by the Kaplan-Meier analysis, using the log Rank (Mantel-Cox) test. Source data are provided as a Source Data file.

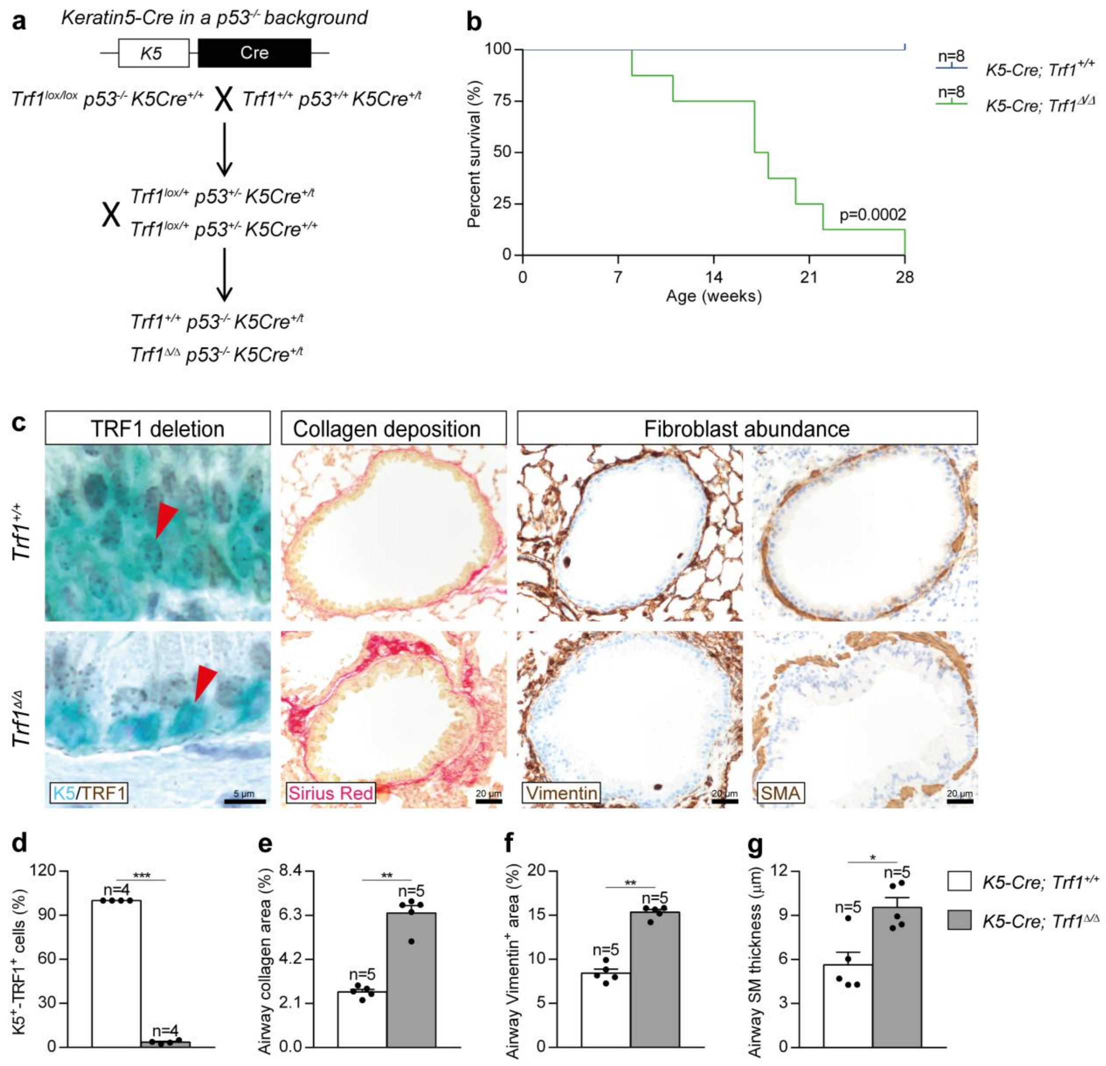

Supplementary Fig. 3 Deletion of *Trf1* in embryonic basal cells decreases survival and increases airway remodeling postnatally. a Generation of *K5-Cre; Trf1*$^{+/+}$ (*Trf1*$^{+/+}$, controls) and *K5-Cre; Trf1*$^{Δ/Δ}$ (*Trf1*$^{Δ/Δ}$) mice in which *Trf1* was deleted in basal cells from day E 11.5 of embryonic development onwards, using the Cre recombinase driven by the *K5* promoter. **b** Kaplan-Meier survival curves of *K5-Cre; Trf1*$^{+/+}$ (*Trf1*$^{+/+}$) and *K5-Cre; Trf1*$^{Δ/Δ}$ (*Trf1*$^{Δ/Δ}$) mice. **c** Representative immunostainings for K5 (blue) and TRF1 (brown) (red arrowheads indicate K5^{+} basal cells with deletion of TRF1), Sirus Red stainings and immunostainings for Vimentin and SMA (airways) in lung sections from *Trf1*$^{+/+}$ and *Trf1*$^{Δ/Δ}$ mice. **d-g** Quantification of p63^{+}-TRF1^{+} cells (%) **(d)**, airway collagen (Sirius Red) **(e)** and Vimentin **(f)** positive areas (%),

and airway smooth muscle (SM) thickness (SMA) (µm) **(g)** in *Trf1*$^{+/+}$ and *Trf1*$^{\Delta/\Delta}$ mice. Data are expressed as mean ± SEM (the number of mice is indicated in each case). *p<0.05, **p<0.01, ***p<0.001 (Mann-Whitney or unpaired t tests). Animal survival was assessed by the Kaplan-Meier analysis, using the log Rank (Mantel-Cox) test). Source data are provided as a Source Data file.

Supplementary Table 1. Primer sets used for qPCR.

Gene	Accession No.	Forward primer (5´-3´)	Reverse primer (5´-3´)
Ccl12	NM_011331.3	TCCTCAGGTATTGGCTGGAC	GGCTGCTTGTGATTCTCCTG
Col1a1	NM_007742.4	CGGAGAAGAAGGAAAACGAG	CAGGGAAACCACGGCTAC
Col1a2	NM_007743.3	GGAACAAATGGGCTCACTGG	CAAGTCCTCTGGCACCTGTA
Col3a1	NM_009930.2	CCCAACCCAGAGATCCCATT	GGTCACCATTTCTCCCAGGA
Col4a1	NM_009931.2	AAGTGGAAGAGATGGAGCCC	CTTCACCTGTCAAACCTGGC
Col5a1	NM_015734.2	TGACAACCGTGAAAGCCAAG	CTTCCCTGTGTGGTCCTCAT
Col6a1	NM_009933.4	GCTACAATGGATGGCTGGTG	GTTCTGTGTGGGTGGGAGTA
Ifng	NM_008337.4	TTCTTCAGCAACAGCAAGGC	ACTCCTTTTCCGCTTCCTGA
Il1b	NM_008361.3	GCAACTGTTCCTGAACTCAACT	ATCTTTTGGGGTCCGTCAACT
Il4	NM_021283.2	CCTCACAGCAACGAAGAACA	CGAAAAGCCCGAAAGAGTC
Il6	NM_031168.1	ACGGCCTTCCCTACTTCACA	CATTTCCACGATTTCCCAGA
Il10	NM_010548.2	GCCTTATCGGAAATGATCCA	TTTTCACAGGGGAGAAATCG
Il13	NM_008355.3	GCCTCCCCGATACCAAAAT	CTTCCTCCTCAACCCTCCTC
Rn18s	NR_003278.3	ATGCTCTTAGCTGAGTGTCCCG	ATTCCTAGCTGCGGTATCCAGG
Tnf	NM_013693.3	GCCTCTTCTCATTCCTGCTTG	CTGATGAGAGGGAGGCCATT

Supplementary methods

Generation of mutant mouse lines

To conditionally delete *Trf1* in fibroblasts, club and basal cells, heterozygous *Trf1$^{lox/+}$ Col1a2/Scgb1a1/p63-Cre^{ERT2} KFP$^{CAG\text{-}lox\text{-}STOP\text{-}lox}$* mice were crossed with *Trf1$^{+/lox}$* mice to obtain *Trf1$^{+/+}$ Col1a2/Scgb1a1/p63-Cre^{ERT2} KFP$^{CAG\text{-}lox\text{-}STOP\text{-}lox}$* and *Trf1$^{lox/lox}$ Col1a2/Scgb1a1/p63-Cre^{ERT2} KFP$^{CAG\text{-}lox\text{-}STOP\text{-}lox}$* mice. Tamoxifen (TMX) was dissolved in corn oil (Sigma Aldrich) to a concentration of 20 mg/ml and intraperitoneally (i.p.) injected to eight-to 10-week-old male *Trf1$^{+/+}$ KFP$^{+/t}$ Cre$^{+/t}$* and *Trf1$^{lox/lox}$ KFP$^{+/t}$ Cre$^{+/t}$* mice (75 mg/kg) to induce the deletion of *Trf1* in fibroblasts, club and basal cells.

Additionally, we have generated the *Trf1$^{\Delta/\Delta}$ K5-Cre p53$^{-/-}$* mouse model that constitutively express the Cre recombinase specifically in basal epithelial cells from day E 11.5 of embryonic development onwards (Martínez *et al*., 2009, Genes Dev. 23, 2060–2075). A p53-deficient background was used to bypass the perinatal lethality associated to constitutive *Trf1* deletion from embryonic development.

In vivo measurement of lung function

The mice were anesthetized by intraperitoneal injection of 10 µl/g of a ketamine-medetomidine anesthetic combination in saline (75:1 mg/kg respectively). A MiniVent (Harvard Apparatus, Holliston, MA, USA) was connected to the plethysmograph and the tracheal cannula for animal ventilation at 10 ml/kg of tidal volume and 150 breaths per minute. Data were measured by 2 pressure transducers that detect pressure variations in the chamber (flow) and in the tracheal cannula (pressure).

Sample collection and processing

Animals were euthanized by intraperitoneal injection of 10 µl/g of a ketamine-xylazine anesthetic combination in saline (100:10 mg/kg respectively) after lung function assessment.

Serum was obtained by centrifugation at 3000 xg for 10 min at 4 °C and stored at -80°C. On the other hand, bronchoalveolar lavage fluid (BALF) was centrifuged at 10000 rpm for 5 min at 4 °C and the supernatants were stored at -80 °C to subsequently assess total protein concentration in BALF using the Pierce BCA Protein Assay Kit (Thermo Fisher Scientific, Waltham, MA, USA). Hereafter, the BALF pellets were resuspended in 500 µl of ACK Lysing Buffer (Thermo Fisher Scientific) and centrifuged at 10000 rpm for 5 min at 4 °C after 10 min of incubation. The supernatants were discarded and 500 µl of PBS 1X were added to the pellet to prepare the BALF cytospin preparations by centrifugation of the slides at 1500 rpm for 5 min.

Quantification of BALF

Total cell number was counted and expressed as cells/ml of BALF, and differential cell counts were performed on May-Grünwald Giemsa (Sigma-Aldrich)-stained cytospins, counting a minimum of 300 cells per slide. Determination of differential cell counts was performed using standard morphology criteria.

Histopathological analyses and immunostaining

Fiji open source image processing software package v1.48r (http://fiji.sc) was used for the quantification of collagen (Sirius Red) and Vimentin, SMA, E-cadherin and Collagen I positive areas (percentage of

DAB), as well as to assess smooth muscle thickness (SMA) and epithelium length measurements. Quantifications in lung sections were performed in 4 different fields or bronchi, respectively in a random way.

Statistics

Statistical analyses were accomplished using SPSS Statistics Software v21 for Windows (IBM, Armonk, NY, USA). For all analyses, a p value<0.05 was considered statistically significant. Results are shown as mean values ± standard error of the mean (SEM). For all analyses, a P value < 0.05 was considered statistically significant.
