# Supplementary material for: Consequences of telomere dysfunction in fibroblasts, club and basal cells for lung fibrosis development
Source: Nat Commun. 2022 Oct 6;13:5656. doi: 10.1038/s41467-022-32771-6 (PMC9537293; doi:10.1038/s41467-022-32771-6)
Supplement: Supplementary file 3 — Reporting Summary [file 41467_2022_32771_MOESM3_ESM.pdf]

nature portfolio

Corresponding author(s): María A. Blasco

Last updated by author(s): Jul 21, 2022

Reporting Summary

Nature Portfolio wishes to improve the reproducibility of the work that we publish. This form provides structure for consistency and transparency in reporting. For further information on Nature Portfolio policies, see our Editorial Policies and the Editorial Policy Checklist.

Statistics

For all statistical analyses, confirm that the following items are present in the figure legend, table legend, main text, or Methods section.

n/a	Confirmed	
☐	☒	The exact sample size (n) for each experimental group/condition, given as a discrete number and unit of measurement
☐	☒	A statement on whether measurements were taken from distinct samples or whether the same sample was measured repeatedly
☐	☒	The statistical test(s) used AND whether they are one- or two-sided<br>*Only common tests should be described solely by name; describe more complex techniques in the Methods section.*
☒	☐	A description of all covariates tested
☐	☒	A description of any assumptions or corrections, such as tests of normality and adjustment for multiple comparisons
☐	☒	A full description of the statistical parameters including central tendency (e.g. means) or other basic estimates (e.g. regression coefficient) AND variation (e.g. standard deviation) or associated estimates of uncertainty (e.g. confidence intervals)
☐	☒	For null hypothesis testing, the test statistic (e.g. F, t, r) with confidence intervals, effect sizes, degrees of freedom and P value noted<br>*Give P values as exact values whenever suitable.*
☒	☐	For Bayesian analysis, information on the choice of priors and Markov chain Monte Carlo settings
☒	☐	For hierarchical and complex designs, identification of the appropriate level for tests and full reporting of outcomes
☒	☐	Estimates of effect sizes (e.g. Cohen's d, Pearson's r), indicating how they were calculated

Our web collection on statistics for biologists contains articles on many of the points above.

Software and code

Policy information about availability of computer code

Data collection: Fiji open source image processing software package v1.48r (http://fiji.sc) was used for the quantification of collagen (Sirius Red) and Vimentin, SMA, E-cadherin and Collagen I positive areas (percentage of DAB), as well as to assess smooth muscle thickness (SMA) and epithelium length measurements.

Data analysis: IBM SPS Statistics 21

For manuscripts utilizing custom algorithms or software that are central to the research but not yet described in published literature, software must be made available to editors and reviewers. We strongly encourage code deposition in a community repository (e.g. GitHub). See the Nature Portfolio guidelines for submitting code & software for further information.

Data

Policy information about availability of data

All manuscripts must include a data availability statement. This statement should provide the following information, where applicable:

- Accession codes, unique identifiers, or web links for publicly available datasets
- A description of any restrictions on data availability
- For clinical datasets or third party data, please ensure that the statement adheres to our policy

The authors declare that data supporting the findings of this study are available within the paper (and its supplementary information files). Source data are provided with the paper.

Field-specific reporting

Please select the one below that is the best fit for your research. If you are not sure, read the appropriate sections before making your selection.

☒ Life sciences ☐ Behavioural & social sciences ☐ Ecological, evolutionary & environmental sciences

For a reference copy of the document with all sections, see nature.com/documents/nr-reporting-summary-flat.pdf

Life sciences study design

All studies must disclose on these points even when the disclosure is negative.

Sample size	The number of mice used was chosen sufficiently large to reach statistical significance if so. No computational analysis was used under the experimental design. We followed the three Rs guiding principles for more ethical use of animal testing
Data exclusions	Not data were excluded from the analysis
Replication	All experiments were performed at least three independent times and/or with sufficient cells/animals per group to demonstrate statistical significance.
Randomization	The mice were randomly allocated within the groups (controls and TRF1 deficient mice)
Blinding	Investigators were blinded to group allocation during experiments. Investigators performing experiments (tamoxifen and bleomycin treatments, measurement of lung function, sample collection and processing, histopathological analyses, IHC, IFC, telomere Q-FISH analyses, RNA isolation, reverse transcription, qPCR and ELISAS) were blinded to controls and TRF1 deficient mice.

Reporting for specific materials, systems and methods

We require information from authors about some types of materials, experimental systems and methods used in many studies. Here, indicate whether each material, system or method listed is relevant to your study. If you are not sure if a list item applies to your research, read the appropriate section before selecting a response.

Materials & experimental systems

n/a	Involved in the study	
☐	☒	Antibodies
☒	☐	Eukaryotic cell lines
☒	☐	Palaeontology and archaeology
☐	☒	Animals and other organisms
☒	☐	Human research participants
☒	☐	Clinical data
☒	☐	Dual use research of concern

Methods

n/a	Involved in the study	
☒	☐	ChIP-seq
☒	☐	Flow cytometry
☒	☐	MRI-based neuroimaging

Antibodies

Antibodies used	IFC and telomere Q-FISH analyses were performed using the following antibodies: COL1A2 (Clone E-6, sc-393573, 1:400, Santa Cruz Biotechnology, Dallas, TX), SCGB1A1/CC10 (Clone E-11, sc-365992, 1:100, Santa Cruz Biotechnology), p63 (Clone 4A4, 790-4509, Roche, Basel, Switzerland), TRF1 (Clone 572C, CNIO Monoclonal Antibodies Core Unit, Madrid, Spain) and 53BP1 (NB100-304, 1:500, Novus Biologicals, Centennial, CO). IHC was performed using the following antibodies: Turbo-RFP (KFP) (AB233, 1:3000, EVROGEN, Moscow, Russia), COL1A2 (Clone E-6, sc-393573, 1:100, Santa Cruz Biotechnology), H2AX (Ser139, 05-636, Clone JBW301, 1:200, EMD Millipore, Burlington, MA), p16 (Clone 33B 1:30, CNIO Monoclonal Antibodies Core Unit), p21 (Clone 291H/B5 1:10, CNIO Monoclonal Antibodies Core Unit), C3 (Asp175, 9661, 1:300, Cell Signaling Technology, Danvers, MA), Ki-67 (Clone D3B5, 12202, 1:50, Cell Signaling Technology), SCGB1A1/CC10 (Clone T-18, sc-9772, 1:1000, Santa Cruz Biotechnology), TRF1 (Clone 572C, ab192629, 1:50, Abcam, Cambridge, UK), p63 (Clone 4A4, 790-4509, Roche, Basel, Switzerland), SOX2 (Clone C70B1, 3728, 1:75, Cell Signaling Technology), Myeloperoxidase (1:1250, GA51161-2, DAKO, Jena, Germany), CD4 (Clone D7D2Z, 25229, 1:50, Cell Signaling Technology), CD8 (Clone 94A 1:200, CNIO Monoclonal Antibodies Core Unit), F4/80 (Clone A3-1, MCA497 1:20, AbD Serotec/Bio-Rad, Hercules, CA), Collagen I (1:600, AB765P, EMD Millipore), Vimentin (Clone D21H3, 5741, 1:50, Cell Signaling Technology), E-cadherin (Clone 36 1:1000, 610181, BD Biosciences) and SMA (Clone 1A4 1:4, GA61161-2, DAKO, Agilent technologies, Santa Clara, CA).
Validation	Validation of antibodies are available in the websites of the folowing companys: Santa Cruz Biotechnology, Roche, Novus Biologicals, EVROGEN, EMD Millipore, Cell Signaling Technology, Abcam, DAKO, AbD Serotec/Bio-Rad, BD Biosciences and Agilent technologies. In the case of antibodies generated by the CNIO Monoclonal Antibodies Core Unit, validation is available in the CNIO Histopathology Unit Repository.

Animals and other organisms

Policy information about studies involving animals; ARRIVE guidelines recommended for reporting animal research

Laboratory animals	Mouse musculus, C57BL/6 background (8-10 weeks old male and female mice)
Wild animals	This study does not involve wild animals
Field-collected samples	This study does not involve samples collected from the field
Ethics oversight	All experiments and animal procedures were approved by our Institutional Animal Care and Use Committee (IACUC) (IACUC.011-2018, CBA_20_2018), by the Ethics Committee for Research and Animal Welfare (CEIyBA) (CBA 20-2018) from the Instituto de Salud Carlos III and by Consejería de Medio Ambiente, Administración Local y Ordenación del Territorio (Comunidad de Madrid) (PROEX 163/18). All experiments and animal procedures were performed in accordance with the guidelines stated in the International Guiding Principles for Biomedical Research Involving Animals, developed by the Council for International Organizations of Medical Sciences (CIOMS). The animals were bred and maintained under specific pathogen-free (SPF) conditions in laminar flow caging at the CNIO animal facility in accordance with the recommendations of the Federation of European Laboratory Animal Science Associations (FELASA).

Note that full information on the approval of the study protocol must also be provided in the manuscript.
